# Simultaneous detection of arboviruses by a multiplex RT-qPCR assay in Tocantins, a northern state of Brazil

**DOI:** 10.1016/j.bjid.2024.103855

**Published:** 2024-07-22

**Authors:** Matheus Martins Daude, Erika Regina Manuli, Geovana Maria Pereira, Alfredo Ramon Alfonso Cavalcante Junior, Ueric José Borges de Souza, Gessi Carvalho de Araujo, Flávio Augusto de Pádua Milagres, Ester Cerdeira Sabino, Horllys Gomes Barreto

**Affiliations:** a Universidade Federal do Tocantins, Faculdade de Medicina, Laboratório de Análise Molecular, Departamento de Ciências da Vida, Palmas, TO, Brazil; b Universidade de São Paulo, Faculdade de Medicina, Instituto de Medicina Tropical, São Paulo, SP, Brazil; c Universidade Federal do Tocantins, Faculdade de Medicina, Instituto de Ciências Biológicas, Palmas, TO, Brazil; d Universidade Federal do Tocantins, Laboratório de Bioinformática e Biotecnologia, Campus Gurupi, Gurupi, TO, Brazil

**Keywords:** Coinfection, Dengue, Zika virus, Quantitative PCR

## Abstract

In Brazil, Dengue, Zika and Chikungunya viruses constitute a major threat to the public health system. Simultaneous circulation of these arboviruses occurs in many regions of the world due to the expansion of transmission vectors. The infection by these arboviruses triggers similar symptoms during their acute phase. However, in some cases, severe symptoms may occur, leading to different types of disabilities and even death. In this context, considering the similarity of the symptoms, the problems caused by the infection of these arboviruses, and the increasing risk of coinfection in humans, the differential diagnosis of these infections is essential for clinical management and epidemiological investigation. Thus, this study aimed to identify, through diagnosis via Quantitative Polymerase Chain Reaction with Reverse Transcription, arbovirus coinfection in patients from the Tocantins state (Northern Brazil). A total of 495 samples were analyzed, three from which were determined to be a coinfection of Dengue and Chikungunya viruses. The data obtained here indicate the co-circulation and coinfection by Dengue and Chikungunya viruses in the Tocantins state. These results highlight the importance of monitoring the circulation of these arboviruses for the development of health actions that aim their prevention and combat, as well as their clinical and therapeutic management.

Arboviruses comprise a group of viruses characterized by their transmission via arthropods and their wide geographic distribution.^1^ The most widespread and prevalent arboviruses belong to the *Flaviviridae* and *Togaviridae* families and are transmitted by mosquitoes of the *Aedes* genus.^1^^,^^2^ Among them, the Dengue Virus (DENV), the Zika vVirus (ZIKV), and Chikungunya Virus (CHIKV) stand out, since they have the same transmission vector, thus representing, for the past few decades, a threat to the global public health and socioeconomic development.^2^^,^^3^

The Dengue, Zika, and Chikungunya infections are endemic, being mainly present in tropical and subtropical regions of the world.^3^ In Brazil, especially in its tropical regions, the *Flaviviridae* and *Togaviridae* families represent the main circulating arboviruses of public health importance,^4^ and DENV, ZIKV and CHIKV constitute a major threat to its public health system due to the high number of probable cases registered annually in the country, in addition to their presence in most Brazilian states.^5^ In 2022, according to the Brazilian Ministry of Health, from February to August, increases of 184.6 %, 86.9 % and 92.6 % for DENV, CHIKV and ZIKV, respectively, were observed in the number of probable cases (BRASIL, 2022). For DENV, 13,62,125 probable cases were reported, representing an incidence rate of 638.5 cases per thousand inhabitants during this time. In relation to CHIKV, the number of probable cases reached 168,908, leading to an incidence rate of 79.2 cases per 100,000 inhabitants. Regarding ZIKV, from January to September of 2022, 10,501 probable cases were identified, corresponding to an incidence rate of 4.9 cases per 100,000 inhabitants.^5^

During the acute phase of DENV, CHIKV and ZIKV infections, similar symptoms are usually present, such as nonspecific febrile syndromes.^6^ DENV infection, for example, presents symptoms such as fever, headache, musculoskeletal pain, maculopapular rash, and conjunctivitis, and is often confused with CHIKV and ZIKV infections due to their similar symptoms.^7^ However, in some cases, clinical conditions may become worse, leading to more severe disabilities and even death.^1^^,^^8^

Simultaneous circulation of these arboviruses occurs in many regions of the world,^9^ and the constant growth of global trade and international travels contributes to this fact, spreading the transmission vectors and, consequently, the arboviruses.^10^ Transmission vector sharing (*Aedes aegypti* and *Aedes albopictus*) is one of the main factors for co-circulation and coinfection of arboviruses.^11^ Thus, the concomitant prevalence and coinfection by these arboviruses have been reported more frequently in several parts of the world^9^^,^^12^, ^13^, ^14^ and, in some cases, this is associated with increases in mortality rates. In Brazil, the co-circulation of DENV, ZIKV and CHIKV represents a potential risk for coinfection and since 2015. It constitutes a challenge for its public health system, not only for the diagnosis, but also for the control of the transmission vector and the clinical and therapeutic management.^15^

In this context, due to the similarity of the initial manifestation of the infections, and consequently the difficulty in differentiating the infection of these arboviruses only by the symptoms they trigger, the increasing risk of coinfection, and also the health potential risk caused by their infection, the differential and accurate diagnosis of these diseases is of fundamental importance for their clinical and epidemiological management.^16^ Thus, the objective of this study was to identify, through diagnosis RT-qPCR (Quantitative Polymerase Chain Reaction with Reverse Transcription) diagnosis, arboviruses coinfection in patients from a north region (the Brazilian state of Tocantins) of Brazil.

In order to be enrolled in this study, patients should have had fever in the last 7 days and be clinically classified as suspected cases of classical dengue, dengue with warning signs, or chikungunya. For classical dengue, symptoms included fever along with at least one of the following symptoms: headache, pain behind the eyes, muscle pain (back, thighs, calves, arms), joint pain, swelling, signs of arthritis, skin rash, or conjunctivitis. Dengue with warning signs is characterized by intense symptoms, including continuous abdominal pain, persistent vomiting, low blood pressure, blurred vision, sweating or fainting upon standing up, mucosal bleeding, excessive drowsiness, mental confusion, impaired speech, or abrupt platelet drop. Similar to classical dengue, chikungunya symptoms included headache, pain behind the eyes, muscle pain (back, thighs, calves, arms), joint pain, joint swelling/redness, signs of arthritis, skin rash, and conjunctivitis.

Patients were invited by the research assistant to participate in the study. After signing the Informed Consent Form (ICF), they answered a questionnaire about their clinical symptoms and provided blood samples for arboviruses diagnosis through RT-qPCR. The biological material was collected at the North Emergency Care Unit (UPA-North) in Palmas, the capital of the Tocantins state, from 05/05/2022 to 09/30/2022. The biological material consisted of 800 µL of blood serum from patients with suspected arboviral infection. The samples used were collected up to the 7th day after the onset of symptoms and stored at −80 °C until RNA extraction. The experiment was carried out at the Molecular Analysis Laboratory (LAM-Human Health) of the Federal University of Tocantins (UFT).

Viral RNA from the collected samples was extracted using the Bio Gene Extraction kit for Viral DNA/RNA (Quibasa/Bioclin), following the manufacturer's instructions. The samples were quantified in a spectrophotometer (Nandrop® Spectrophotometer) at A260, and all samples showed a high degree of purity and satisfactory concentration (> 50 ng/µL). After RNA extraction, samples were stored at −80 °C until diagnosis via RT-qPCR.

RT-qPCR reactions were performed using the one-step TaqMan™ Arbovirus Triplex Kit (ZIKV/DENV/CHIKV – ThermoFisher Scientific) and the TaqMan™ Arbovirus Triplex Control Kit (ZIKV/DENV/CHIKV – ThermoFisher Scientific) was used as a positive control, according to the manufacturer's instructions from both kits. The amplification reactions were carried out using an ABI PRISM 7500 Fast Real-Time PCR thermal cycler (Applied Biosystems). Data were collected and stored in the 7500 Fast Software (Version 2.1).

The identified coinfection cases were submitted to genome sequencing. Extracted RNA was converted to cDNA by using the Luna Script RT SuperMix (5×) (New England Biolabs, Ipswich, MA, USA). The synthesized cDNAs were used as templates for whole-genome amplification by multiplex PCR using the CHIKV and DENV sequencing primers’ scheme (divided into two separated pools), which were designed as previously described by Quick et al., 2017.^17^

The MinION library preparation was performed using the Ligation Sequencing Kit SQK-LSK-109 and the Native Barcoding Kit EXP-NBD104 (Oxford Nanopore, Technologies, Oxford, United Kingdom). The resulting library was loaded on a R9.4.1 MinION Flow Cell (FLO-MIN106D) and sequenced using a MinION Mk1B device. The FASTQ reads generated with Nanopore sequencing were mapped and assembled by using the ARTIC bioinformatics Medaka pipeline (Version 1.0.3, Oxford Nanopore Technologies, Oxford, United Kingdom). The consensus genomes were assigned to genotypes using the Genome Detective Typing Tool.^18^

A total number of 495 samples were collected from 05/05/2022 to 09/30/2022. The diagnosis of DENV, ZIKV and CHIKV via RT-qPCR was performed on all collected samples, allowing the detection of 123 positive samples for CHIKV, 30 positive samples for DENV, and three samples displayed a coinfection of DENV and CHIKV. The Clinical manifestations of the three patients displaying coinfection are described in Table 1 and, as one can observe, the first patient (220166063001) showed the typical symptoms of Chikungunya infection, while the other two patients (220166063701 e 220166079001) displayed characteristic symptoms of dengue infection.

**Table 1:** Clinical manifestations of the three patients detected with arboviruses coinfection in the northern Brazilian state of Tocantins from 05/05/2022 to 09/30/2022.

Patient	Age (years) and gender	Symptoms
220166063001	26, female	Fever, headache, muscle pain (back, thighs, calves, arms), joint pain, swelling, signs of arthritis (inflammation of the joints) and skin rash.
220166063701	26, female	Fever, headache, muscle pain (back, thighs, calves, arms).
220166079001	58, female	Fever, headache, muscle pain (back, thighs, calves, arms), pain behind the eyes, low blood pressure, blurred vision, sweating or fainting upon standing up.

The Cq values of the three samples showing a coinfection are described in Table 2. ZIKV was not detected in any of the samples. The controls used validated the assay, guaranteeing the reliability of the results (Table 2). The DENV and CHIKV amplification curves for the three co-infected samples are shown in the Supplementary Material S1. For these samples, Cqs values were below 35 (Table 2) and the RT-qPCR characteristic exponential amplification curves were observed for all of them.

**Table 2:** Cq (Cycle of quantification) values obtained via RT-qPCR for the samples showing a coinfection (DENV and CHIKV) and for the controls used in the assay.

Sample	Cq (DENV)	Cq (CHIKV)	Cq (C.I)	Cq (C.P)	Cq (C.N)
220166063001	33.58	16.19	30.74	D 27.95; C 28.64	S.A
220166063701	33.1	17.3	29.14	D 27.95; C 28.64	S.A
220166079001	21.99	32.3	28.8	D 30.88; C 35.26	S.A

DENV or D, Dengue Virus; CHIKV or C, Chikungunya virus; C.I, Internal Control; C.P, Positive Control; C.N, Negative Control; S.A, No Amplification.

By using a portable MinION sequencer and an amplicon approach it was generated, for samples 220166063001 and 220166063701, a partial and near-complete genomes only for CHIKV with a coverage of 95.6 % and 91.2 %, respectively (Table 3). For DENV it was not possible to recover a partial genome with enough coverage, probably due to the high Cqs values observed for these samples. For the sample 220166079001, it was recovered partial genomes for CHIKV and Dengue virus serotype 1 (DENV-1), with coverage means of 86.7 % and 81.1 %, respectively (Table 3). The three CHIKV genomes belonged to East/Central/South Africa (ECSA), which has been reported as the predominant genome in Brazil as a whole and also in Tocantins.^19^ The DENV genome belonged to genotype V.

**Table 3:** Data associated with the sequenced samples analyzed in this study.

Sample	Accession ID	Coverage	Depth of Coverage	Virus	Lineage
220166063001	OR538602	95.6	9025.6	CHIKV	ECSA
220166063701	OR538603	91.2	3826.8	CHIKV	ECSA
220166079001	OR538604	86.7	510.3	CHIKV	ECSA
220166079001	OR523627	81.1	791.0	DENV-1	Genotype V

The co-circulation and coinfection by DENV, ZIKV and CHIKV has been the subject of several studies, considering the serious problems that they represent.^9^^,^^12^, ^13^, ^14^ In 2014, the main Chikungunya epidemic that impacted the Caribbean and South America raised concerns about the frequency of simultaneous arbovirus infections. In this same year, a study carried out in Haiti, comprising 100 children who had an undifferentiated acute febrile illness, identified 82 positive cases for CHIKV, from which six and one showed a coinfection with ZIKV and DENV, respectively.^14^ In Colombia, a wide study from 2018, which aimed to determine the frequency of coinfection by DENV, CHIKV and ZIKV, during October 2015 to December 2016, showed that from a total of 23,871 samples, 34 displayed a coinfection, CHIKV-ZIKV (28 samples), DENV-CHIKV (three samples), and DENV-ZIKV (three samples).^9^ In other parts of the world, such as Yemen, Gabon and India, the co-circulation and coinfection of these arboviruses has also been observed.^15^^,^^20^^,^^21^

In Brazil, the same scenario of co-circulation and coinfection observed in other parts of the world has also been reported in different studies.^12^^,^^13^^,^^15^^,^^22^ In the Brazilian state of Bahia, in 2016, 15 patients with a presumptive diagnosis of an acute viral disease were evaluated by next-generation metagenomic sequencing, and ZIKV/CHIKV coinfection was found in two patients.^22^ In another region of Brazil (Pernambuco state), in the same year, an DENV outbreak occurred, where 77 patients suspected of DENV infection were evaluated by RT-qPCR, sequencing and ELISA (Enzyme-linked Immunosorbent Assay), in order to identify the viral infection, with two samples being found to be positive for DENV and ZIKV.^13^ In Tocantins, an RT-qPCR study with 102 samples from patients with symptoms compatible with arboviral infection showed that five patients displayed a coinfection of CHIKV and DENV.^12^ These studies indicate that coinfection cases are relatively rare compared to the total number of monoinfections, even in endemic areas. This pattern can be accentuated when research is conducted during periods of lower circulation of the mosquito vectors (and, consequently, of the viruses), as observed in this study, in which samples were collected during the dry season of the Tocantins state.

Studies aiming to detect co-circulation and coinfection by arboviruses have gained notable attention from researchers in the past few years, mainly due to the difficulty of differentiating single and coinfections, due to their similar clinical aspects,^23^ and also due to the problems caused by coinfection with arboviruses.^9^ Simultaneous infection by DENV and CHIKV has been associated with more severe symptoms, reinforcing the need for a better understanding of the pathogenesis of this association.^9^^,^^21^ Some studies have linked ZIKV-CHIKV coinfection with cases of fetal mortality.^9^ In addition, the ZIKV-CHIKV coinfection has been associated to fatal adult cases, with an association to the development of neurological symptoms and nosocomial infections.^9^ DENV-CHIKV coinfection has also been linked to fatal cases.^21^

Studies aiming to monitor the simultaneous circulation and identifying the coinfection by arboviruses are thus essential, and the data obtained here corroborate to those previously described in the literature and demonstrate the co-circulation and coinfection by DENV and CHIKV in the state of Tocantins, similar to what has been found in other regions of Brazil and parts of the world. Although ZIKV infection was not identified in any sample from this study, monitoring the circulation of these arboviruses is important for the development of health actions focused in the prevention, combat, and clinical and therapeutic management. Thus, this study can support the development of health actions in the state of Tocantins, given the alert that the results obtained can generate for the public and private health system.

## Conflicts of interest

The authors declare no conflicts of interest.

## Appendix Supplementary materials

Image, application 1
