# Supplementary material for: Simultaneous detection of arboviruses by a multiplex RT-qPCR assay in Tocantins, a northern state of Brazil
Source: Braz J Infect Dis. 2024 Jul 22;28(4):103855. doi: 10.1016/j.bjid.2024.103855 (PMC11345543; doi:10.1016/j.bjid.2024.103855)

**BJID-D-23-00302 - Supplementary Material S1**

Supplementary Figure 1 Amplification curves of the sample 660630, identified as displaying more than one arbovirus. The straight lines indicate the threshold. (A) DENV, Dengue Virus; (B) CHIKV, Chikungunya Virus; (C) PPIA (Internal Control).


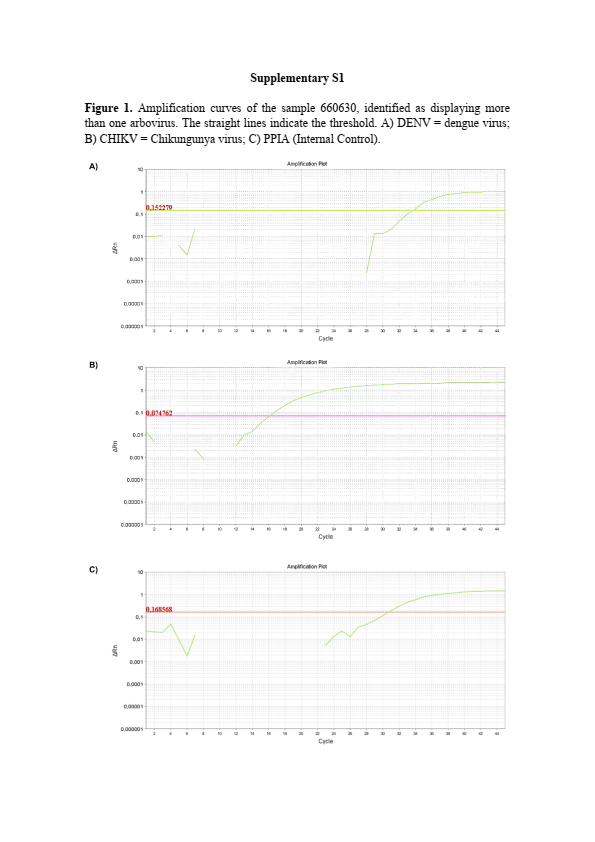


Supplementary Figure 2 Amplification curves of the sample 660637, identified as displaying more than one arbovirus. Straight lines indicate the threshold. (A) DENV, Dengue Virus; (B) CHIKV, Chikungunya Virus; (C) PPIA (Internal Control).


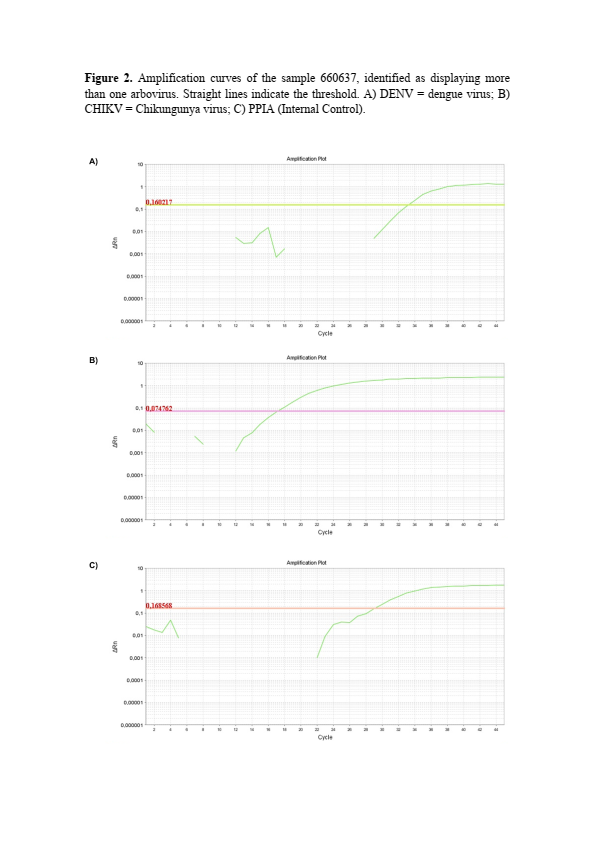


Supplementary Figure 3 Amplification curves of the sample 660790, identified as displaying more than one arbovirus. Straight lines indicate the defined threshold. (A) DENV, Dengue Virus; (B) CHIKV, Chikungunya Virus; (C) PPIA (Internal Control).


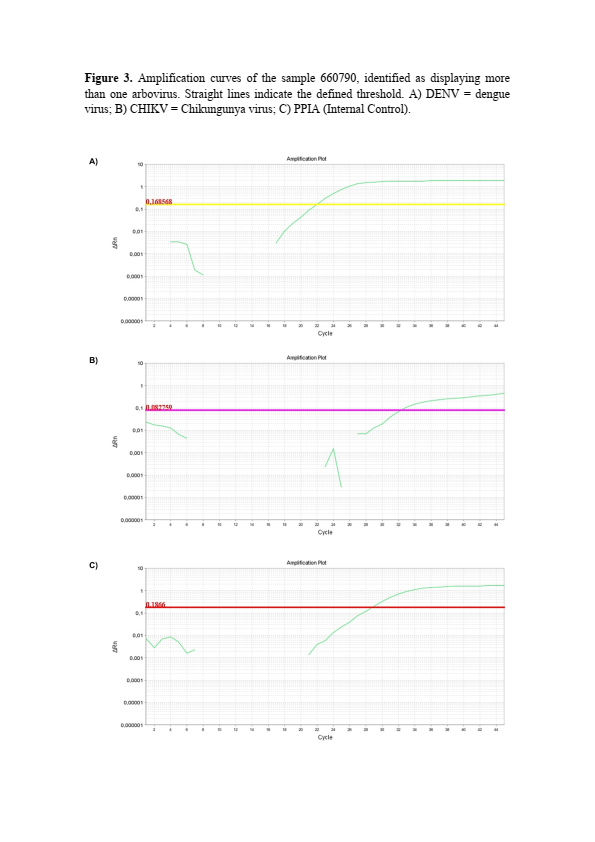

Supplement: Supplementary file 1 [file mmc1.docx]
